# Surgical management of multiple rib fractures in polytrauma patients: semi-damage control surgery

**DOI:** 10.7150/ijms.102790

**Published:** 2024-11-04

**Authors:** Shuhuan Li, Chu Wang, Pan Hu, Tingmin Xu, Bo Chen, Feifei Jin, Diya Sun, Tianbing Wang, Wei Huang

**Affiliations:** 1 Trauma Treatment Center, Peking University People's Hospital, Beijing, P.R. China.; 2 Key Laboratory of Trauma Treatment and Neural Regeneration (Peking University) Ministry of Education; National Center for Trauma Medicine, Beijing 100044, P.R. China.

**Keywords:** polytrauma, rib fracture, timing of surgery, damage control surgery

## Abstract

**Background**: To investigate the timing and extent of surgery for rib fractures in polytrauma patients.

**Methods**: Data from polytrauma patients who underwent early and partial rib fracture fixation after successful resuscitation were retrospectively analyzed. The study encompassed demographic data, clinical data, and outcomes.

**Results**: In total, 71 patients with polytrauma were included. ISS ranged from 16 to 50 with a mean score of 25.3±7.5. The median lactate level was 3.6 mmol/L (IQR: 3.1 to 4.5), the median base deficit (BD) was 8.2 mmol/L (IQR: 6.4 to 9.8) and the shock index (SI) median was 1.2 (IQR: 0.9 to 1.3). Total fractured ribs in 71 patients were 726; individually, the minimum and maximum number of fractured ribs was 3 and 22, respectively (median, 10; mean, 10.2 ± 4.0). The average time to surgery was 42.9±42.6 h. Specifically, 41(57.7%) received the surgery within 24h and 52 (73.2%) patients received the surgery within 3 days following successful resuscitation. A total of 246 (33.9%) ribs underwent open reduction and internal fixation with plate, 3.46 ribs for each patient, with high frequencies of the 6th (49, 19.9%), 5th (46, 18.7%), 4th and 7th ribs (both 36, 14.6%). The average length of ICU stay was 11.5 ± 7.5 days and the duration of hospitalization was 16.3 ± 9.9 days. No surgical site infection or mortality was observed.

**Conclusions:** Early and partial rib fracture fixation to restore the relative stability of the thorax is safe and effective for polytrauma patients after successful resuscitation. This surgery strategy is called semi-damage control surgery.

## Introduction

Chest trauma is the leading cause of death among patients with trauma, accounting for approximately 35% of trauma-related mortality 1. Respiration can be adversely affected following chest trauma, with potential damages to the airways or lungs (e.g., pulmonary contusion) and the mechanical structure of breathing (e.g., ribs). This can lead to severe pain, hypoventilation, hypoxemia, impaired expectoration, and an increased risk of pneumonia. In patients with fractured ribs, the overall mortality can reach 10% 2, 3.

Non-surgical treatment strategies for rib fractures include adequate analgesia, respiratory support, such as intubation and tracheotomy when necessary. However, conservative treatment often shows limited effectiveness 4. According to data from the National Trauma Data Bank, in the early 21st century, non-surgical treatment remained the primary option for managing rib fractures, with less than 5% of patients receiving surgical stabilization of rib fractures (SSRF) 5. In recent years, with advances in internal fixation materials and surgical techniques, SSRF has gained increasing attention from clinicians. The application of SSRF has resulted in a substantial reduction in mortality compared to the adoption of non-surgical treatment. A meta-analysis of eight randomized controlled studies showed that surgical treatment could significantly reduce pneumonia, ICU time, and mechanical ventilation time 6. For simple chest trauma, rib fixation surgery is usually performed within 2 - 3 days after injury 7-9. However, polytrauma patients may also present with hemorrhagic shock, severe craniocerebral injury, coagulation disorders or infection at the early stage, which may affect the surgical plan for rib fractures. The timing and extent to perform SSRF remain not entirely clear. Therefore, this study retrospectively analyzed the early and partial fixation strategy for rib fractures at our center, aiming to provide insights into the appropriate timing and surgical approach for managing multiple rib fractures in polytrauma patients.

## Methods

### Study design

This was a retrospective case analysis study and included polytrauma patients who received rib operation after successful resuscitation from September 2019 to October 2023. The patient's Injury Severity Score (ISS) was greater than or equal to 16, with conditions including flail chest, non-flail chest with severe chest wall deformity, or three or more ipsilateral severely displaced rib fractures 8, 10. The exclusion criteria were as follows: non-polytrauma, aged <18 or >90 years, with a history of malignancy, or transferred to our hospital after being hospitalized in another hospital for more than 3 days. This study received ethical approval from the Ethics Review Committee of Peking University People's Hospital (approval number: 2024PHB207-001).

Polytrauma was defined as the presence of severe injuries with an Abbreviated Injury Scale (AIS) score ≥ 3 in more than two different anatomical divisions combined with changes in one or more of the following pathological parameters: systolic blood pressure ≤ 90 mmHg, Glasgow Coma Scale (GCS) score ≤ 8, base excess (BE) ≤ -6, International Normalized Ratio (INR) ≥ 1.4 or Activated Partial Thromboplastin Time (APTT) ≥ 40 s, and age ≥ 70 years 11. In this study, base deficit (BD) was used instead of base excess (BE), with BD being the negative value of BE, commonly applied in trauma assessment.

Successful resuscitation was defined as achieving an arterial blood gas (ABG) lactate level ≤ 2 mmol/L with hemodynamic stability. The period between the time of successful resuscitation and time of SSRF is defined as the “time to surgery”.

Surgery was performed early after successful resuscitation, provided the patient did not have irreversible traumatic brain injury, high cervical spinal cord injury, or other injuries or comorbidities that required long-term mechanical ventilation, or if the patient or their authorized family members refused rib surgery after successful resuscitation. The need for long-term mechanical ventilation was evaluated by two attending doctors. All included patients undergoing open reduction of rib fractures and internal fixation using plates and screws. In the operation, we selected to fix partial rib fractures and palpated the chest wall to confirm whether the thorax had restored relative stability.

### Data collection and variables

Data collection encompassed information on gender, age, injury mechanism, ISS, chest AIS score, history of cardiopulmonary diseases (e.g., heart failure, heart infarction, chronic obstructive pulmonary disease (COPD), interstitial pneumonia), number of fractured ribs, incidences of pulmonary contusion, pneumothorax, hemothorax, hemopneumothorax, hospitalization duration, admission to intensive care unit (ICU), length of ICU stay, tracheal intubation, tracheotomy, time of successful resuscitation, time of SSRF, rib segments involved in surgery, surgical site infection, mortality, and reasons for time to surgery more than 72 h. Upon admission, vital signs such as systolic blood pressure (SBP), heart rate (HR), respiratory rate (RR), and Glasgow Coma Scale (GCS) were recorded. Additionally, data on lactate levels, base deficit (BD), shock index (SI), and blood transfusion requirements were collected.

### Statistical analysis

Statistical methods: the description of quantitative indicators will calculate the mean, standard deviation, median, minimum, maximum, lower quartile (Q1), upper quartile (Q3), and interquartile range (IQR). Categorical indicators will describe the number of cases and composition ratios of each type. The statistical software is Microsoft Excel Version 2019.

## Results

Forty-five cases were excluded, including 28 non-polytrauma patients, 13 patients who were transferred from other hospitals more than 3 days, 4 patients with a history of malignancy. A total of 71 polytrauma patients were included in this study, including 51 males and 20 females with a mean age of 57.3 ± 13.1 years (range, 23-84 years). Upon admission to the emergency department (ED), the median SBP was 93 mmHg (IQR: 81 to 105), with 49.3% of patients presenting with an SBP below 90 mmHg, and 9.9% with an SBP below 70 mmHg. The median HR was 102 bpm (IQR: 96 to 109), and the median RR was 26 breaths per minute (IQR: 23 to 28). The median GCS score was 9 (IQR: 8 to10). The median lactate level was 3.6 mmol/L (IQR: 3.1 to 4.5), and the median BD was 8.2 mmol/L (IQR: 6.4 to 9.8). The median SI was 1.2 (IQR: 0.9 to 1.3). ISSs ranged from 16 to 50 with a mean score of 25.3 ± 7.5 (mean± standard deviation). The average total blood transfusion volume for all patients was 11.7 units (± 8.6), which included an average of 8.4 units (± 6.0) of packed red blood cells (PRBC), 3.3 units (± 2.7) of fresh frozen plasma (FFP), and 0.2 units (± 0.9) of platelets (Table 1).

**Figure 1:**
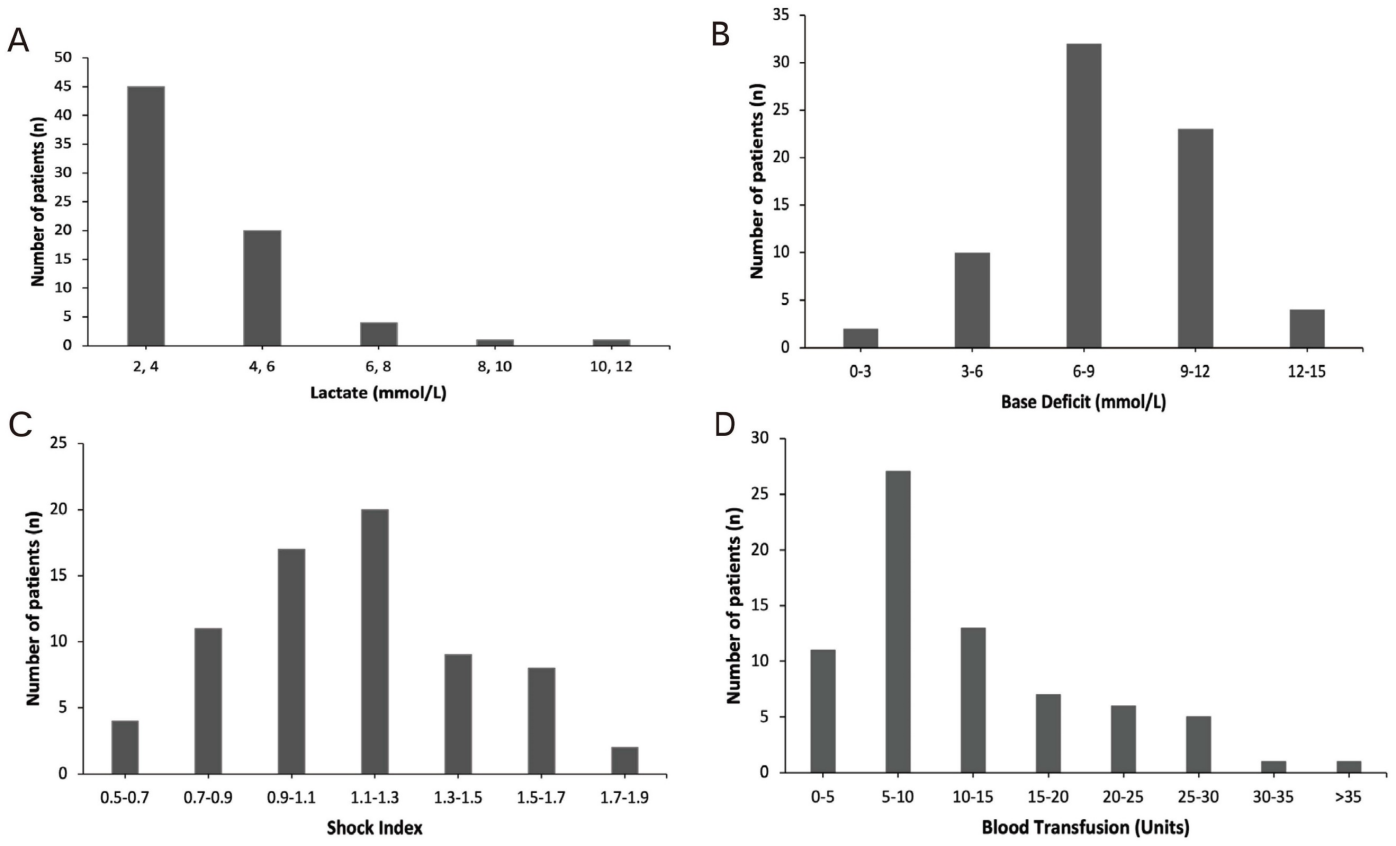
Distribution of (A) lactate (mmol/L); (B) base deficit (BD); (C) shock index (SI); and (D) total blood transfusion requirements (packed red blood cells + fresh frozen plasma + platelets) in patients.

For most patients on admission, lactate was between 2 and 4, with 45 patients in this range (Figure 1A), BD was between 6 and 9 observed in 32 patients (Figure 1B), SI was between 1.1 and 1.3 for 20 patients (Figure 1C). During hospitalization, 27 patients needed 5-10 units blood (Figure 1D).

The causes of injury included traffic incidents in 43 cases (60.6%), falls from height in 16 cases (22.5%), falls in 7 cases (9.9%), struck by objects in 3 cases (4.2%), injuries by machinery in 1 case (1.4%), and methane gas explosion in 1 case (1.4%). The total number of fractured ribs in 71 patients was 726. For an individual patient, the minimum number of fractured ribs was 3 and maximum was 22, with a median of 10 and mean of 10.2 ± 4.0 (mean ± standard deviation). 13 (18.3%) patients had paradoxical respiration. All patients had pulmonary contusion, among whom 35 had hemopneumothorax, 16 had hemothorax, 6 had pneumothorax, and the rest 14 had no hemo/pneumothorax.

The average duration of hospitalization was 16.3 ± 9.9 days. 67 (94.4%) patients required treatment in ICU and the average length of ICU stay was 11.5 ± 7.5 days. 52 (73.2%) patients required endotracheal intubation and mechanical ventilation support. Pneumonia occurred in 8 patients (11.2%) during hospitalization, and acute kidney injury (AKI) was reported in 10 patients (14.8%). 5 (7.0%) patients underwent tracheotomy. No incidence of chest surgical site infection (SSI) or mortality was observed during hospitalization (Table 2).

**Figure 2:**
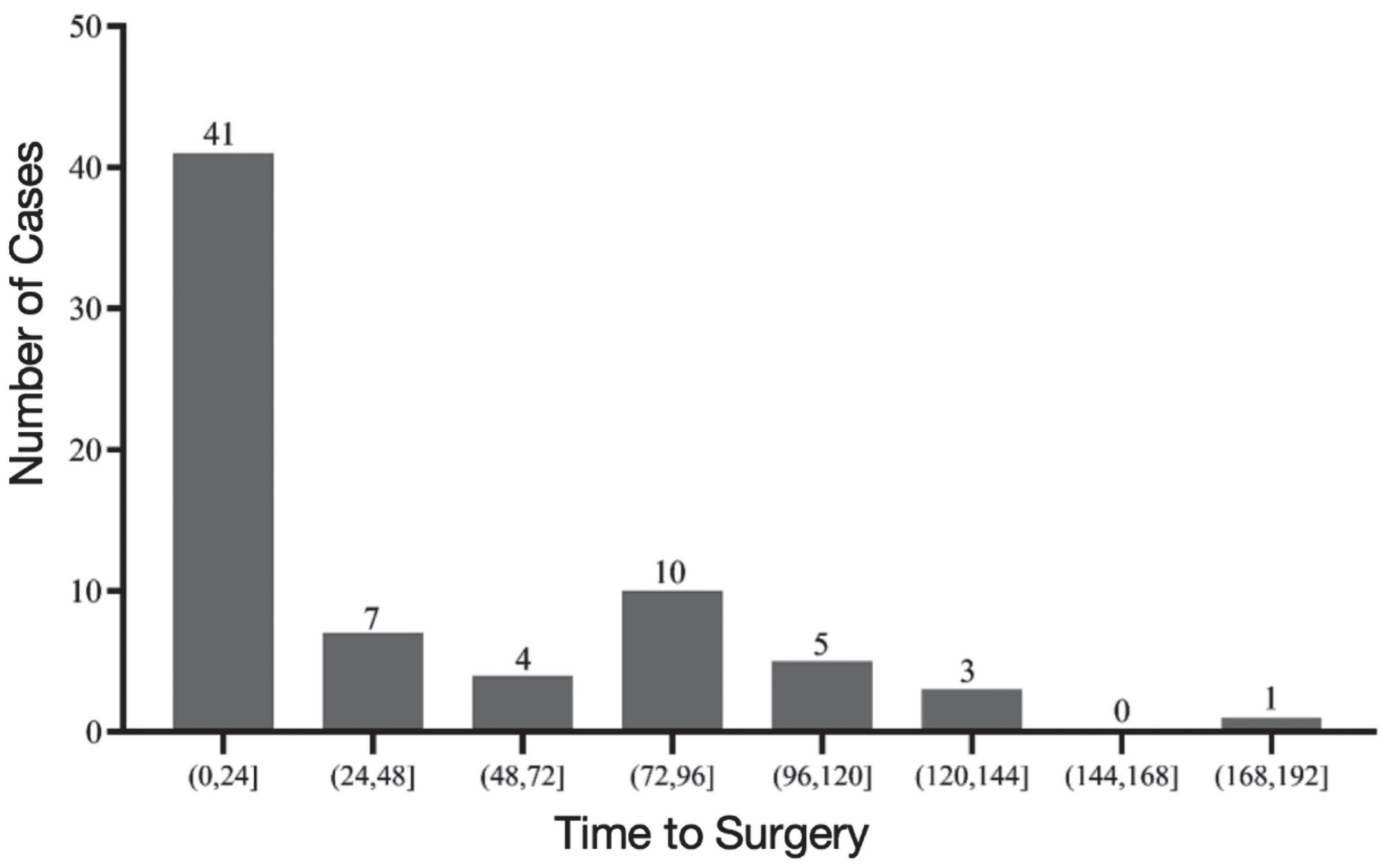
Distribution of time to surgery.

The average time to surgery was 42.9 ± 42.6 h. Specifically, 41(57.7%) received the surgery within 24h and 52 (73.2%) patients received the surgery within 3 days following successful resuscitation (Figure 2). Whereas, 19 (26.8%) patients received SSRF more than 3 days following successful resuscitation (Figure 2). Reasons for time to surgery more than 3 days included concurrent operations with other surgeries in 10 cases (14.8%), stabilization of other combined organ injuries (brain injury, aortic dissection and maxillofacial injury) in five cases (7.0%), re-bleeding after successful resuscitation in one case (1.4%), controlling abdominal infection in one case (1.4%) and due to surgical resource allocation in two cases (2.8%).

**Figure 3:**
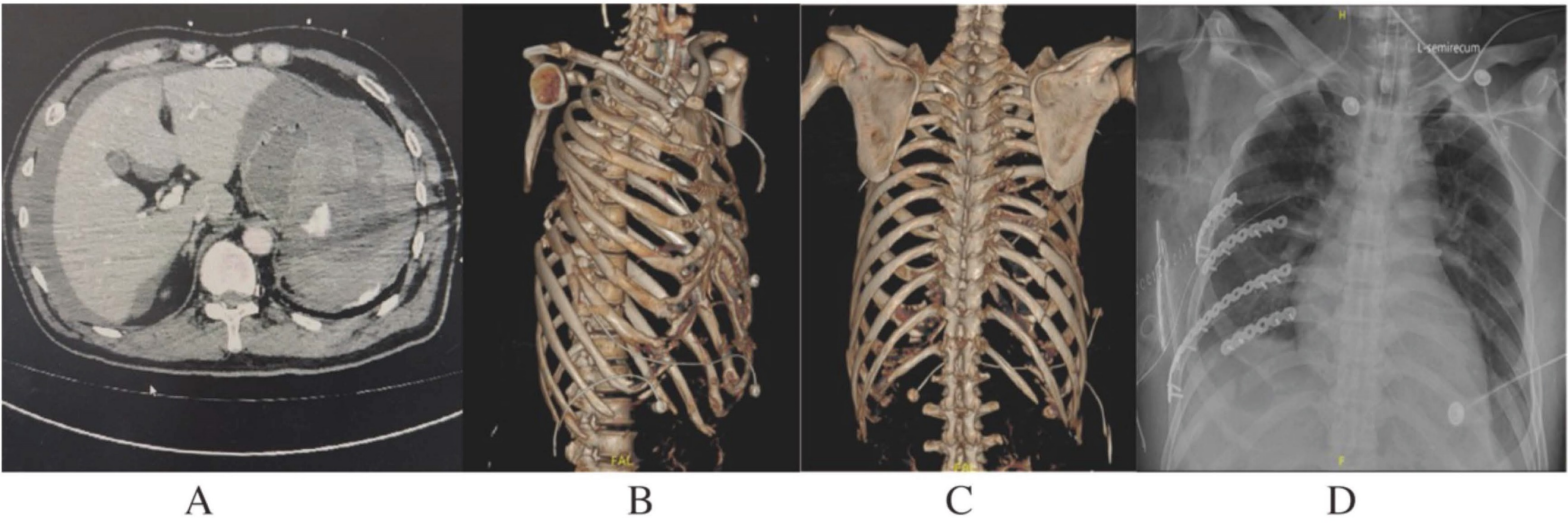
A Polytrauma Patient with Splenic Rupture and Multiple Rib Fractures. A 52-year-old male sustained fall-from-height injury with an ISS of 25, diagnosed with hemorrhagic shock, splenic rupture, and multiple rib fractures. A, contrast-enhanced CT scanning revealed a Grade V splenic rupture with contrast extravasation. B, C, the 3rd to 10th rib fractures with severe chest wall deformity of right side. On admission, the patient underwent an emergency exploratory laparotomy and splenectomy and was transferred to the ICU for resuscitation. D, 20 hours after successful resuscitation, partial ribs (5th to 8th) were fixed to restore the relative stability of the chest.

A total of 246 (33.9%) ribs underwent open reduction and internal fixation with plate, 3.46 ribs for each patient, with the 6th (49, 19.9%), 5th (46, 18.7%), 4th (36, 14.6%), and 7th (36, 14.6%) ribs being the most frequently surgically fixed (Table 3). Figure 3 showed a typical case.

## Discussion

Decisions regarding the timing for performing SSRF and determining the extent of surgery in polytrauma patients are challenging in clinical practice. We select successful resuscitation (lactate level ≤ 2 mmol/L with hemodynamic stability) as the timing for SSRF. Surgical strategy was partial rib fixation to restore relative stability of thorax. During current study, the time to surgery was within 24h in nearly 60% patients and within 72 hours in more than 70% patients. Only one-third of the fractured ribs were fixed to restore the relative stability of the thorax. No mortality was observed in this setting. The findings indicated that early SSRF to fix partial fractures was safe and effective for polytrauma patients after successful resuscitation. This treatment strategy is termed as semi-damage control surgery.

The application of surgical treatment for rib fractures has increased remarkably 12. Surgery increased by 76% from 2007 to 2014 and that the mortality of surgical group was notably lower than non-surgical treatment (1.58% VS 5.3%) in American 5. For patients with severe trauma in the chest wall with an AIS score ≥ 3, the mortality in the surgical group was significantly lower than that in the non-surgical group (3.8% vs. 8.6%) 13. Meta-analysis and randomized clinical trials also showed that morbidity and mortality were significantly higher in the nonoperative group 14-16. Therefore, surgical treatment of rib fractures had gradually gained the attention of clinicians. However, polytrauma patients might also present with shock, multiple organ dysfunction, infection, fever, coagulation disorders, and unstable organ injuries in the early stage, which may affect the fixation of rib fractures. The timing of surgery is challenging to determine and necessitates a comprehensive risk-benefit analysis to determine the optimal timing.

Compared with other studies on polytrauma patients with multiple rib fractures undergoing SSRF, our study's ISS score of 25.3 ± 7.5 is similar with the ISS score of 27 (SD 11.2) reported in another research. Other studies report an ICU stay of 16.1 ± 15.7 days and a total hospital stay of 29.3 ± 18.7 days, whereas in our study, the ICU stay was 11.5 ± 7.5 days, and the total hospital stay was 16.3 ± 9.9 days. These data suggest faster recovery in our patient group without an increased risk of complications 17. A randomized controlled trial on surgical rib fixation versus nonoperative management in severe chest wall injury reported an ISS score of 22 (17-32) in the surgical group, which is lower than that of our patient cohort. Their study reported an ED heart rate of 93 (22) bpm, respiratory rate of 21 (5) breaths/min, and ED systolic blood pressure of 125 (30) mmHg. In contrast, our study showed a mean heart rate of 102 (96,109) bpm, respiratory rate of 26 (23,28) breaths/min, and ED systolic blood pressure of 93 (81,105) mmHg, indicating more severe conditions in our patients with a higher shock burden. Furthermore, our pneumonia incidence was 11.2%, lower than their 21% 18.

### Time of surgery: successful resuscitation

Polytrauma is considered "severe" when the ISS is ≥ 16 and often requires damage control surgery (DCS) to stabilize the systemic physiopathological conditions, mainly complete hemostasis and control of contamination 19. Respiratory dysfunction or failure due to flail chest or severe chest wall deformity can be addressed by mechanical ventilation at the early stage and does not require DCS. However, mechanical ventilation is not a definitive treatment for multiple rib fractures, the morbidity and mortality tend to increase with prolonged mechanical ventilation 20. Also, the delay of SSRF will also compromise overall patient care and impede the definitive treatment of other injury such as pelvic, spine injury and so on.

Both the “Consensus Statement: Surgical Stabilization of Rib Fractures Rib Fracture Colloquium Clinical Practice Guidelines (2017)” 4 in the United States and the “The Chinese Consensus for Surgical Treatment of Traumatic Rib Fractures 2021” 21 recommend that rib fixation surgery be performed within 72 h of the injury for multiple rib fractures. Wang *et al.* 9 concluded from a multicenter randomized controlled trial that surgery within 48 h post-injury was effective in reducing hospitalization duration, length of ICU stays, and duration of mechanical ventilation. Bethlahmy *et al.* 7 recommended the surgery for managing multiple rib fractures to be performed within 2-3 days post-injury. According to the study by Patel DD *et al.* 22, in patients with flail chest, early surgical intervention (within 4 days) significantly reduces pulmonary complications. Simmonds A *et al.* 23 found that patients with flail chest without severe head trauma who underwent SSRF within 3 days of injury experienced significantly reduced morbidity. Even if there was traumatic brain injury, early SSRF within 72 hours was recommended 24. In polytrauma patients, SSRF is feasible but it should not interfere with the treatment of emergent visceral injuries, and it can be delayed with satisfactory postoperative outcomes 25. Therefore, to optimize outcomes, surgery for multiple rib fractures should be performed early, preferably within 72 h of the injury. However, in polytrauma patients, determining the optimal timing for rib surgery remains inconclusive due to the need for simultaneous management of other injuries and the possibility to sustain early respiratory function through mechanical ventilation.

After achieving definitive hemostasis and controlling contamination, further efforts should focus on optimizing hemodynamics, correcting acid-base imbalances, improving coagulation function, and enhancing microvascular perfusion. The main clinical indicators of effective circulation and sufficient microvascular perfusion include the level of ABG lactate 26. Ouellet *et al.* 27 found a significant increase in mortality in patients with polytrauma with lactate level > 2.2 mmol/L. Most studies set the lactate threshold at 2-2.5 mmol/L, with a few others setting it at 3-4 mmol/L 28. Based on the literature, we set a lactate level of less than or equal to 2 mmol/L as the threshold for early successful resuscitation, which is usually accomplished within the first 24-48h 19. We believe that the time of achieving successful resuscitation is appropriate for considering surgical treatment of rib fractures. In this study, nearly 60% patients underwent SSRF within 24h after successful resuscitation. Compared with the existing literature, our surgical treatment was more aggressive and the timing of surgery was earlier. However, 26.8% patients received SSRF more than 3 days, this reflected the complexity of treatment in polytrauma patients. The timing of SSRF should be tailored to patient's concomitant injuries, such as brain injury, rebleeding or infection.

### Extent of surgery: partial rib stabilization

Despite significant advances in materials and techniques for rib fracture fixation, not all ribs are easily accessible for surgical treatment. Due to the difficult exposure of ribs 1-3, and their minimal impact on respiratory function, surgical treatment is rarely performed. Surgical treatment is necessary only if the floating ribs are associated with liver or spleen injury. The main principles of definitive surgical treatment of rib fractures recommended in the literature include: the fractures on the 1st, 2nd, 11th, and 12th ribs generally do not need surgical fixation, whereas the other displaced ribs should be consecutively fixed, with all fracture sites fixed to avoid pain due to rib shifts associated with respiratory motion 4, 21. But rib fracture surgery itself can also cause side injury to patients. Polytrauma patients were critically ill, the surgical management of rib fractures may not be exactly the same as the principles of definitive surgery. Therefore, it is very important to seek a balance between the fixation effect and surgical damage.

The 4th to 10th ribs are the most mobile ribs and mainly upheld the chest wall and generate most obvious pain 29, 30. The 6th-8th ribs greatly impact respiratory function 31, 32. Additionally, our previous study showed that the displacement and collapse of the 6th to 8th lateral ribs had the greatest impact on chest volume and surgical fixation of these ribs might be more important for restoring chest volume 32. For flail chest, there was no difference in narcotic use, chest wall deformities, or lung function between partial and complete rib stabilization. So, partial rib stabilization was an acceptable strategy 33. Sebastian Reindl *et al.* 34 used thoracoscopy to evaluate the stability of the osteosynthesis and concluded that it was not necessary to stabilize every fracture, especially posterior and paravertebral rib fractures. Not every fractured rib requires fixation, not every fracture of the same rib requires treatment and paravertebral fractured rib rarely require stabilization 35. Intraoperative synchronous palpatory assessment the stability of the chest wall plays a crucial role.

The scapula is attached to the posterior-lateral aspect of the chest, spanning the 2nd to 7th ribs. On the one hand, the scapula can provide some stability to the ribs. On the other hand, surgical fixation of the ribs under the scapula results in extensive soft tissue dissection, causing significant injury to the patient. Therefore, we rarely fix the ribs under the scapula.

In this study, there were 726 fractured ribs, with 246 ribs getting fixed and the 5th-6th ribs being the most frequently fixed. Not every displaced rib fracture was fixed consecutively. This corresponds with the findings of Fokin AA *et al.*, who indicated that rib fractures are typically stabilized from the third to the eighth ribs 36. Similarly, Wen-Ruei Tang and colleagues had noted that the most frequently surgically stabilized ribs were the fourth to the eighth ribs 37.

### Semi-DCS

Damage control techniques have been utilized by surgeons for over a century. It was not until the 1980s that DCS began to take shape 38. DCS has been applied in abdominal, thoracic and vascular trauma 39. Damage Control Orthopedics (DCO) is a strategy focused on managing and stabilizing major orthopedic injuries in selected polytrauma patients who are in an unstable or severely compromised physiological condition. Its primary objectives are to control hemorrhage, temporarily stabilize major skeletal fractures, and manage soft tissue injuries while minimizing surgical trauma to the patient 40. Rib fractures are associated with increased mortality and significant morbidity in polytrauma patients 41. Regarding to the time for surgery, we don't need to conduct DCS or DCO for rib fractures, as the polytrauma patients are often hemodynamically unstable and patients' respiratory function can be improved and maintained through the use of ventilators in the early stages. Regarding to the extent for surgery, not every displaced rib fracture needs to be fixed, the relative stability of the thorax should be achieved with minimal injury. This early and partial fixation of rib fractures after successful resuscitation is termed as semi-DCS.

There was zero post-operative mortality suggesting that performing SSRF was safe and effective under principle of semi-DCS in polytrauma patients. The critically ill patients which failed to achieve hemodynamic stability during resuscitation, or had conditions such as irreversible traumatic brain injuries, severe abdominal infection or high cervical spinal cord injury requiring long-term mechanical ventilation, were not suitable for SSRF and didn't include in this research. This was also one of the important reasons for the low mortality rate observed in this study.

### Limitations of the study

We acknowledge several important limitations to our study that must be kept in mind when interpreting our results. Firstly, this is a single-center retrospective case analysis and just a summary of our experience and lacks a control cohort, potentially limiting a comprehensive evaluation of the surgery's efficacy. Secondly, for different doctors, there may be bias in the selection of surgical patients, especially when excluding unsuitable surgical patients. Additionally, the study population consisted of patients with varying degrees of injury severity and comorbidities, which may have impacted the evaluation of surgical outcomes. For example, severe head trauma or other organ injuries could affect both the timing and effectiveness of rib fracture stabilization surgery. Consequently, subgroup analyses will be necessary in future studies to thoroughly understand the potential effects of these factors on surgical outcomes. In the future, we will design multicenter retrospective case-control study and prospective multicenter randomized controlled study to further validate the effectiveness of this surgical strategy.

## Conclusions

Early and partial rib fracture fixation to restore the relative stability of the thorax is safe and effective for polytrauma patients after successful resuscitation. This surgery strategy is called semi-damage control surgery.

## Figures and Tables

**Table 1:** Demographics, ED Vital Signs, and Transfusion Data

Variable	Total (N=71)
Age, Mean (± SD)	57.3 (± 13.1)
Male, n (%)	51 (71.8)
Female, n (%)	20 (28.2)
ED first vital signs	
SBP, Median (Q1, Q3)	93 (81,105)
<90 mmHg, n (%)	35 (49.3)
<70 mmHg, n (%)	7 (9.9)
HR, Median (Q1, Q3)	102 (96,109)
RR, Median (Q1, Q3)	26 (23,28)
GCS, Median (Q1, Q3)	9 (8,10)
Lactate, Median (Q1, Q3)	3.6 (3.1,4.5)
BD, Median (Q1, Q3)	8.2 (6.4,9.8)
SI, Median (Q1, Q3)	1.2 (0.9,1.3)
ISS, Mean (± SD)	25.3 (± 7.5)
Total blood Transfusion (units), Mean (± SD)	11.7 (± 8.6)
PRBC (Units), Mean (± SD)	8.4 (± 6.0)
FFP (Units), Mean (± SD)	3.3 (± 2.7)
Platelets (Units), Mean (± SD)	0.2 (± 0.9)

SD: Standard deviation; ED: Emergency department; SBP: Systolic blood pressure; HR: Heart rate; RR: Respiratory rate; GCS: Glasgow coma scale; BD: Base deficit; SI: Shock index; ISS: Injury severity score. PRBC: Packed red blood cells; FFP: Fresh frozen plasma.

**Table 2:** The outcomes of polytrauma patients underwent SSRF

Clinical outcomes overview	Total (N=71)
Mortality, n (%)	0
HLOS days, Mean (± SD)	16.9 (± 9.9)
ICULOS days, Mean (± SD)	11.5 (± 7.5)
Pneumonia, n (%)	8 (11.2)
AKI, n (%)	10 (14.8)
Tracheotomy, n (%)	5 (7)
Chest SSI	0

HLOS: Hospital length of stay; ICULOS: Intensive care unit length of stay; AKI: Acute kidney injury; SSI: Surgical site infection.

**Table 3:** The number and proportion of ribs were fixed for each rib

Rib	No. of surgery (n)	Proportion (%)
1st	0	0
2nd	0	0
3rd	24	9.7
4th	36	14.6
5th	46	18.7
6th	49	19.9
7th	36	14.6
8th	33	13.4
9th	16	6.5
10th	6	2.4
11th	0	0
12th	0	0
Total	246	100
